# Resetting the ligand binding site of placental protein 13/galectin-13 recovers its ability to bind lactose

**DOI:** 10.1042/BSR20181787

**Published:** 2018-12-14

**Authors:** Jiyong Su, Linlin Cui, Yunlong Si, Chenyang Song, Yuying Li, Tong Yang, Hao Wang, Kevin H. Mayo, Guihua Tai, Yifa Zhou

**Affiliations:** 1 Jilin Province Key Laboratory for Chemistry and Biology of Natural Drugs in Changbai Mountain, School of Life Sciences, Northeast Normal University, Changchun 130024, China; 2 Department of Biochemistry, Molecular Biology & Biophysics, 6-155 Jackson Hall, University of Minnesota, 321 Church Street, Minneapolis, MN 55455, U.S.A.

**Keywords:** Galectin-13, Galectin-13 cellular distribution, Ligand binding site reconstruction, Placental protein 13, Site-directed mutagenesis

## Abstract

Placental protein 13/galectin-13 (Gal-13) is highly expressed in placenta, where its lower expression is related to pre-eclampsia. Recently, the crystal structures of wild-type Gal-13 and its variant R53H at high resolution were solved. The crystallographic and biochemical results showed that Gal-13 and R53H could not bind lactose. Here, we used site-directed mutagenesis to re-engineer the ligand binding site of wild-type Gal-13, so that it could bind lactose. Of six newly engineered mutants, we were able to solve the crystal structures of four of them. Three variants (R53HH57R, R53HH57RD33G and R53HR55NH57RD33G had the same two mutations (R53 to H, and H57 to R) and were able to bind lactose in the crystal, indicating that these mutations were sufficient for recovering the ability of Gal-13 to bind lactose. Moreover, the structures of R53H and R53HR55N show that these variants could co-crystallize with a molecule of Tris. Surprisingly, although these variants, as well as wild-type Gal-13, could all induce hemagglutination, high concentrations of lactose could not inhibit agglutination, nor could they bind to lactose-modified Sepharose 6b beads. Overall, our results indicate that Gal-3 is not a normal galectin, which could not bind to β-galactosides. Lastly, the distribution of EGFP-tagged wild-type Gal-13 and its variants in HeLa cells showed that they are concentrated in the nucleus and could be co-localized within filamentary materials, possibly actin.

## Introduction

Galectin (Gal) is a lectin that binds to β-galactosides [1]. Until now, 13 members of the galectin family (Gal-1, -2, -3, -4, -7, -8, -9, -10, -12, -13, -14, -16, and -17A) in humans have been identified [2,3]. Based on their folded monomer and quarternary structures, they have been classified into three types: chimera, tandem repeat, and prototype [4]. Gal-1, -2, -7, -10, -13, -14, -16, and -17A belong to prototype group [3,4]. Crystallographic and biochemical studies have shown that prototype galectins could form dimeric structures [5–9]. However, with the exception of Gal-1 and -2, their quarternary dimeric structures vary significantly from each other [8,9].

We first solved the crystal structure of Gal-13, whose original name was placental protein 13 [10]. Because the primary and tertiary structures of Gal-13 are highly conserved among all galectins, it had been named galectin-13 [11,12]. However, our crystallographic and biochemical experiments (solid phase experiment, hemagglutination assay, and biolayer interferometry) proved that wild-type Gal-13 could not bind lactose or a rhamnogalacturonan Ι domain (RG-I-4) from Ginseng Pectin [9]. This finding runs contrary to previous solid phase experiments showing that Gal-13 could bind lactose-modified agarose beads [11]. Primary structure alignment showed that a highly conserved histidine residue was modified to arginine (Arg53) in the ligand binding site of wild-type Gal-13, which may be why Gal-13 could not bind lactose. In order to recover the lactose binding ability of Gal-13, we generated an R53H variant. However, this variant still could not bind lactose, but it could be co-crystallized with glycerol [9], implying that other residues within the Gal-13 ligand binding site may play roles in lactose binding. Upon further analysis of the amino acid sequence and 3D structure of Gal-13, we identified several residues that were likely responsible for the inability of Gal-13 to bind lactose. We then mutated them to demonstrate their importance in ligand binding and to recover the ability of Gal-13 binding to lactose. If we could recover the ability of Gal-13 to bind lactose, then this would mean that wild-type Gal-13 is not a normal galectin.

The gene of Gal-13 was only identified in Gal-13 primates. It plays a key role in pregnancy of primates. During pregnancy, Gal-13 levels in maternal serum continuously increase, and near term, its concentration is approximately three times higher than in the serum of non-pregnant women. Post-partum, Gal-13 vanishes from maternal blood within 2–5 weeks [13]. A reduced Gal-13 mRNA level in placentas from the first and third trimesters has been assumed to indicate HELLP syndrome and pre-eclampsia [14,15]. Therefore, the Gal-13 concentration in maternal serum may be useful for predicting pre-eclampsia [16]. Immunofluorescence and immunohistochemistry experiments showed Gal-13 is predominantly expressed in the placenta (specifically in the syncytiotrophoblast) [17]. However, the published immunological results of the distribution of Gal-13 in cells were not consistent. Several results showed that Gal-13 was only distributed in the cytoplasm and on the cell membrane [3,17–21], whereas other results showed that Gal-13 could also be localized in the nucleus [22,23]. Our previous results showed that Gal-13 is primarily distributed in the nucleus [9]. In addition, immunological experiments showed that Gal-13 could be co-localized with annexin II and actin in the syncytiotrophoblast [17,18]. However, the use of EGFP-tagged Gal-13 to study its distribution in cells has not been investigated, and it is unknown which residues in the ligand binding site are important for Gal-13 distribution.

Here, in order to discover why Gal-13 cannot bind lactose and to try to recover its ability to do so, we generated several variants that contain two to four mutations within the ligand binding site. We then co-crystallized the variants with lactose, and performed hemagglutination and solid phase assays to investigate the ability of these variants to bind lactose. Finally, we investigated the distribution of EGFP-tagged Gal1-3 variants in HeLa cells.

## Materials and methods

### Cloning, expression, and purification of Gal-13

The wild-type Gal-13 overexpression construct (pET28a_Gal-13) was used as the site-directed mutation template in this report. Site-directed mutagenesis of Gal-13 was carried out with the QuickChange XL site-directed mutagenesis kit (Stratagene). The mutation plasmids were confirmed by DNA sequencing. *Escherichia coli* BL21 (DE3) cells were transformed with these recombinant plasmids and induced to express proteins by incubating with 0.5 mM IPTG for 16 h at 25°C. The variants were extracted and purified using a Ni-NTA agarose column (Qiagen, Hilden, Germany) according to previously reported protocols [9]. Following purification, the variants were dialyzed in 10 mM HEPES, pH 7.0. During overnight dialysis at 4°C, thrombin was added to remove His tags from the protein. Each milligram of His-tagged protein was digested with 20 units (NIH unit) of thrombin. As determined by sodium dodecyl sulfate-polyacrylamide gel electrophoresis (SDS-PAGE), the purity of the resulting protein was >90%. Finally, variants were concentrated to 5 mg/ml using an Amicon Ultra-15 Centrifugal Filter Unit (3 kDa cut off) and stored at −80°C.

### Crystallization, data collection, and structure determination

To obtain crystals that were suitable for X-ray diffraction, we used the hanging-drop method. Crystals of Gal-13 variants were obtained between 7 and 14 days from drops that contained 1 µl protein and 1 µl solution from the well containing 0.1 M Tris–HCl, pH 8.5, 0.05 M lactose, 0.2 M NaF, 20% (w/v) PEG 3350 at room temperature. Prior to X-ray data collection, the crystals were soaked for approximately 1 min in the reservoir solution supplemented with 20% (v/v) PEG400 as a cryoprotectant and then flash cooled in liquid nitrogen. Data sets were collected at 100 K at the Shanghai Synchrotron Radiation Facility 18U1 (Shanghai, China).Data sets were indexed and integrated using XDS [24] or HKL-3000 [25] and scaled using Aimless [26] from the CCP4 package (6.4.0) [27]. Structures were determined by Phaser [28] with a molecular replacement method using the structure of Gal-13 (PDB: 5XG7) [9] as the search model. Structure refinement and water updating were performed using Phenix [29] refine and manual adjustment. Final structure validations were performed using MolProbity [30,31].

### Hemagglutination assay

The hemagglutination assay was performed as previously reported [9]. Chicken erythrocytes were prepared from fresh blood collected in Alsever’s medium (0.8% sodium citrate, 2.05% glucose, 0.42% sodium chloride, and 0.055% citric acid) and were washed five times with 0.15 M NaCl. Cells were then suspended in PBS (pH 7.4) containing 1 mg/ml trypsin and incubated at 37°C for 1 h. After washing with 0.15 M NaCl, the cells were fixed in PBS buffer (pH 7.4) for 1 h containing 1% glutaraldehyde at room temperature followed by termination with five volumes of 0.1 M glycine in PBS buffer (pH 7.4). The fixed cells were washed and adjusted to 10% (v/v) with PBS buffer (pH 7.4). The hemagglutination assay was performed in microtiter V plates, with each well containing 75 µl variants or 75 µl control solution and 25 µl 4% (v/v) chicken erythrocyte suspensions. The cells were added last, followed by shaking, and agglutination was allowed to proceed for 1 h on the ice to ensure the temperature was consistent.

### Solid phase assay

Lactose–agarose beads was generated according to previous protocol [9]. Variants (25 µg) were added to 50 µl lactose–Sepharose 6b beads. The solution was incubated in 1.5-ml microtubes with gentle stirring at 4°C for 2 h. Tubes were then centrifuged at 1000×***g*** for 1 min to sediment the agarose beads. The beads were washed in buffer containing 135 mM NaCl, 2.7 mM KCl, 1.5 mM KH_2_PO_4_, and 10 mM Na_2_HPO_4_. Bound protein was eluted with 300 mM lactose, sequentially.

### Transfection and fluorescence microscopy

To assess the subcellular localization of Gal-13 variants, we transiently transfected variants into HeLa cells by using PEI as previously reported [9]. The variants were cloned into pEGFP-C2 vector. EGFP was constructed in the N-terminus of variants. HeLa cells were maintained in Dulbecco’s modified Eagle’s medium (DMEM) supplemented with 10% newborn bovine serum and 100 units/ml of penicillin–streptomycin at 37°C in an atmosphere of 5% CO_2_/air. HeLa cells were plated in three wells of a six-well plate in 2 ml complete medium at a density of 2–3×10^5^/ml, and the medium was completely changed before transfection. A volume of 3 μg of plasmid and 3 μg of PEI were diluted, with 300 μl of blank medium per well and mixed completely and incubated for 20 min at room temperature. The mixtures with pEGFP-C2-variants were placed into the wells. After 6 h of transfection, the medium was replaced with complete DMEM and continuously maintained at 37°C in a humidified atmosphere of 5% CO_2_/air. Transfection efficiency was determined by fluorescence microscopy at 16 h at room temperature.

## Results

### Preparation of Gal-13 variants

**Figure 1:**
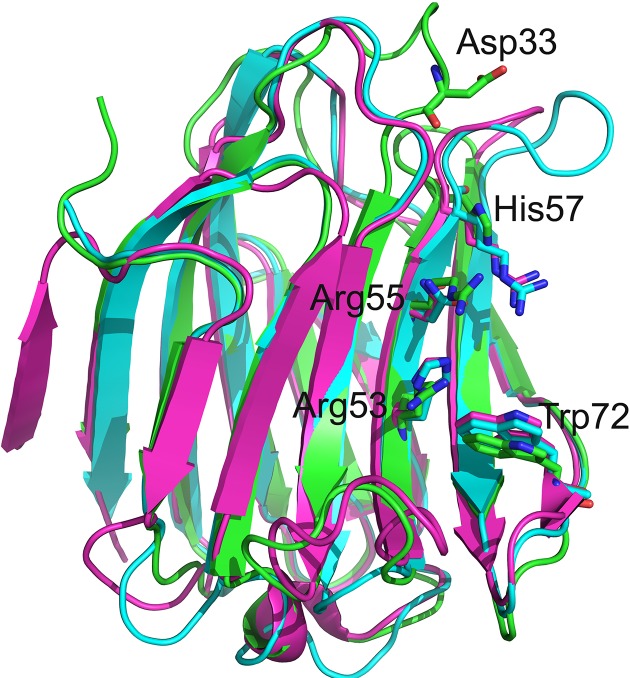
The overlay of wild-type Gal-13 (labeled by green) with the Gal-3 CRD (PDB: 4R9C, labeled by cyan) and Gal-8 N-terminal CRD (PDB: 5GZD, labeled by purple) The corresponding residues of Gal-13 Arg53, Arg55, and His57 in Gal-3 CRD and Gal-8 N CRD are histidine, asparagine, and arginine, respectively. The three residues in the sugar binding sites of Gal-3 CRD and Gal-8 N CRD are important for binding lactose. In the present study, Gal-13 Arg53, Arg55, and His57 were mutated to the corresponding residues in Gal-3 CRD and Gal-8 N CRD (Table 1). Asp33 could insert into the sugar binding site of another Gal-13 monomer in another asymmetric unit. This aspartate residue was also mutated to glycine.

Previously, we generated a primary structure alignment of Gal-13 with other galectins [9]. The alignment showed that several key amino acid residues (Arg53, Arg55, and His57) in the ligand binding site of Gal-13 were not conserved as are residues in Gal-3 and Gal-8N carbohydrate-binding domain (CRD) that bind lactose. Here, we structurally superimposed Gal-13 and Gal-3 [32] and Gal-8N CRD [33] (Figure 1). Gal-13 has Arg53, Arg55 and His57, whereas Gal-3 has His158, Asn160, and Arg162, and Gal-8N has His65, Asn67, and Arg69 in those positions. These three residues in Gal-3 and Gal-8N are crucial for interactions with the galactose residue in lactose. His158 and His65 (located at the same positions as Arg53 in Gal-13) at the bottom of the ligand binding site of Gal-3 and Gal-8 N CRD make important hydrogen bonds with galactose O4 to stabilize the binding [34,35]. Arg162 in Gal-3 and Arg69 in Gal-8N (located at the same position as His57 in Gal-13) also could stabilize the pyran ring of galactose [32,33]. Gal-13 has such a dramatically different ligand binding site compared with Gal-3 and Gal-8N that this protein cannot bind lactose. In order to investigate the importance of these residues in binding lactose and to try to recover the ability of Gal-13 to bind lactose, we systematically generated several variants of Gal-13 (Table 1). Moreover, in the crystal, Asp33 from one monomer occupies part of the ligand binding site of another Gal-13 monomer. In order to eliminate steric hindrance, this aspartate was mutated to glycine in several variants (Table 1). Following the site-directed mutagenesis, we expressed these Gal-13 variants in *E. coli* and concentrated them to 5 mg/ml. In order to better compare structures, the variants were crystallized under similar conditions as previously reported [9].

**Table 1:** Gal-13 variants studied in this report

Protein name	Protein overexpression constructs	EGFP-tagged constructs
Wild-type Gal-13 [9]	pET-28a_Gal-13 [9]	pEGFP-C2_Gal-13 [9]
R53H [9]	pET-28a_R53H [9]	pEGFP-C2_R53H
C136SC138S [9]	pET-28a_C136SC138S [9]	pEGFP-C2_C136SC138S
R53HR55N	pET-28a_R53HR55N	pEGFP-C2_R53HR55N
R53HH57R	pET-28a_R53HH57R	pEGFP-C2_R53HH57R
R53HR55NH57R	pET-28a_R53HR55NH57R	pEGFP-C2_R53HR55NH57R
R53HR55ND33G	pET-28a_R53HR55ND33G	pEGFP-C2_R53HR55ND33G
R53HH57RD33G	pET-28a_R53HH57RD33G	pEGFP-C2_R53HH57RD33G
R53HR55NH57RD33G	pET-28a_R53HR55NH57RD33G	pEGFP-C2_R53HR55NH57RD33G

### Ligand binding sites of Gal-13 variants

The space group of the structures of Gal-13 variants solved here was same as we previously reported for Gal-13 [9]. Structural statistics for the variants are provided in Table 2. Differences in C α RMSD values compared with those reported for other Gal-13 structures (PDB codes: 5XG7, 5XG8, 5Y03) are less than 0.5 Å, indicating that our Gal-13 structures are similar to each other and that site-directed mutagenesis did not influence Gal-13 structures.

**Table 2:** Data collection and refinement statistics

Parameters	R53H	R53HR55N	R53HH57R	R53HH57RD33G	R53HR55NH57RD33G
PDB code	6A66	6A65	6A63	6A62	6A64
Resolution (Å)	19.11–1.40 (1.42–1.40)	18.91–1.77 (1.81–1.77)	18.89–1.63 (1.66–1.63)	18.92–2.03 (2.09–2.03)	18.91–1.63 (1.66–1.63)
Space group	C222	C222	C222	C222	C222
Unit cell parameters (*a, b, c*) (Å)	58.23	57.32	56.93	57.36	57.35
	92.06	91.70	92.96	92.66	92.58
	50.68	50.34	50.52	50.34	50.31
No. of measured reflections	168473 (3870)	13108 (723)	106771 (5452)	55165 (4312)	107070 (5441)
No. of unique reflections	27115 (1248)	13108 (723)	17086 (843)	8973 (690)	17066 (837)
Completeness (%)	99.7 (95.0)	98.7 (93.6)	99.8 (100.0)	99.8 (99.9)	99.9 (100.0)
Multiplicity	6.2 (3.1)	1.0 (1.0)	6.2 (6.5)	6.1 (6.2)	6.3 (6.5)
*R*_merge_ (%)	6.3 (36.8)	15.5 (58.6)	5.7 (48.8)	3.3 (10.1)	4.0 (17.9)
<*I*/δ(*I*)>	12.1 (1.6)	15.7 (3.9)	14.2 (3.1)	26.3 (11.1)	22.5 (7.1)
*R*_model_ (%)	18.40	20.08	17.97	16.36	17.04
*R*_free_ (%)	19.49	25.40	20.89	21.34	20.66
RMSD bond lengths (Å)	0.008	0.006	0.006	0.007	0.006
RMSD bond angles (°)	1.033	0.848	0.905	0.949	0.897
Ramachandran plot residues in favored regions (%)	95.59	95.59	96.32	97.06	96.32
Ramachandran outliers (%)	0.74	0.74	0.74	0.74	0.74
Ligand	Tris	Tris	Lactose	Lactose	Lactose

**Figure 2:**
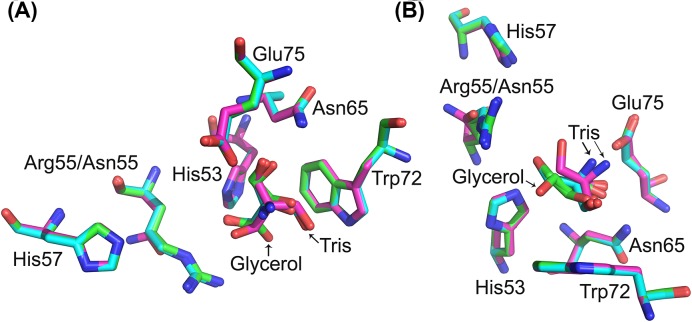
Co-crystallization of R53H and R53HR55N with Tris Tris molecules in R53H and R53HR55N were compared with glycerol molecules in R53H (PDB: 5XG8). (**A**) Top view. (**B**) Side view. Tris molecules in the sugar binding sites show triangle shapes.

In our previous Gal-13 structural study [9], we tried to co-crystallize wild-type Gal-13 with lactose, but failed. We assumed that mutation of Arg53 to histidine would be sufficient to recover the ability of Gal-13 to bind lactose. However, this was not the case, and R53H did not co-crystallize with lactose. However, by soaking the R53H crystal in the cryoprotectant containing 20% (v/v) glycerol, we could solve the R53H crystal structure that contained a molecule of glycerol in two different orientations within the ligand binding site. In the present study, we used PEG400 as the cryoprotectant for R53H crystal, and not glycerol. Surprisingly, we found a different compound bound in the ligand binding site of R53H. Mass spectroscopy (Supplementary Table S1) identifies this compound as Tris (Figure 2). Crystallization for this R53H crystal containing 50 mM lactose is the same as before [9]. However, here, lactose could not co-crystallize with R53H, but Tris could. Tris has three hydroxyl groups and binds in a triangular shape (Figure 2A). Aside from R53H, the double mutant R53HR55N also could co-crystallize with Tris, but not lactose. The Tris molecule in R53HR55N occupies a different position compared with Tris in R53H. It seems that Arg55 plays a role in stabilizing Tris in R53H. In contrast, Asn55 could not stabilize Tris, and its hydroxyl group points up in direction (Figure 2B). The previously solved co-crystal structure of R53H with glycerol was also merged with the two structures solved here. The hydroxyl groups of glycerol in one conformation could almost be merged with the Tris molecule in R53H.

**Figure 3:**
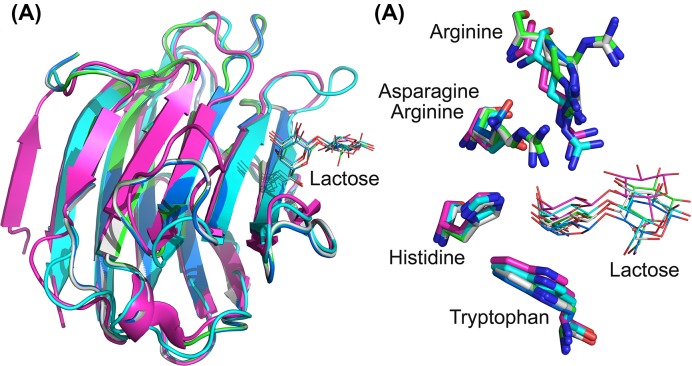
Comparison of three Gal-13 variant structures [R53HH57R (labeled by green), R53HH57RD33G (labeled by white) and R53HR33NH57RD33G (labeled by blue)] with Gal-3 CRD (labeled by cyan) and Gal-8 N CRD (labeled by purple) (**A**) Global overlay. (**B**) The overlay of the sugar binding sites of Gal-13 variants, Gal-3 CRD, and Gal-8 N CRD. In Gal-13 variants, His53, Asn55, and Arg57 in Gal-13 variants show similar conformations to the corresponding residue of Gal-3 CRD and Gal-8 N CRD. Arg57 in R53HH57 and R53HH57RD33G shows two conformations. The galactose residues of lactoses in the sugar binding sites show similar conformations. However, the positions of galactose residues are not consistent.

**Figure 4:**
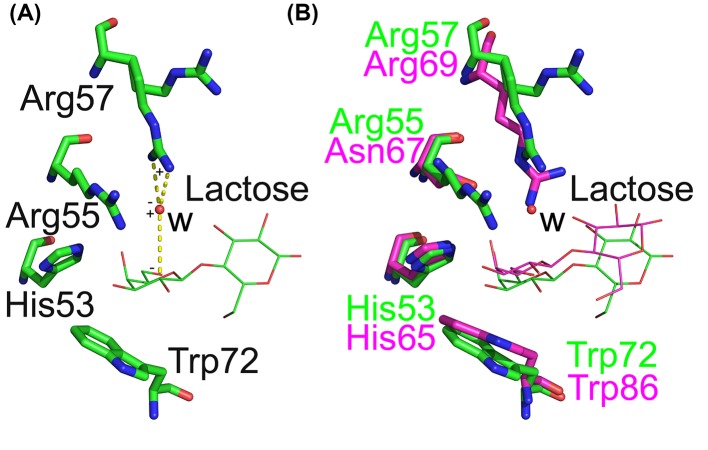
Stabilization of galactose pyran ring by Arg57 (**A**) In R53HH57R, Arg57 could indirectly stabilize the galactose pyran ring through a water molecule (W) by cation–π interaction. (**B**) The overlay of the sugar binding sites of R53HH57R (labeled by green) and Gal-8 N CRD (labeled by purple). W could perfectly merge with a nitrogen atom of guanidine side chain of Gal-8 Arg69, which could stabilize the pyran ring through a cation–π interaction. However, the distance between W and the pyran ring is longer than the distance between the nitrogen atom and the pyran ring.

**Figure 5:**
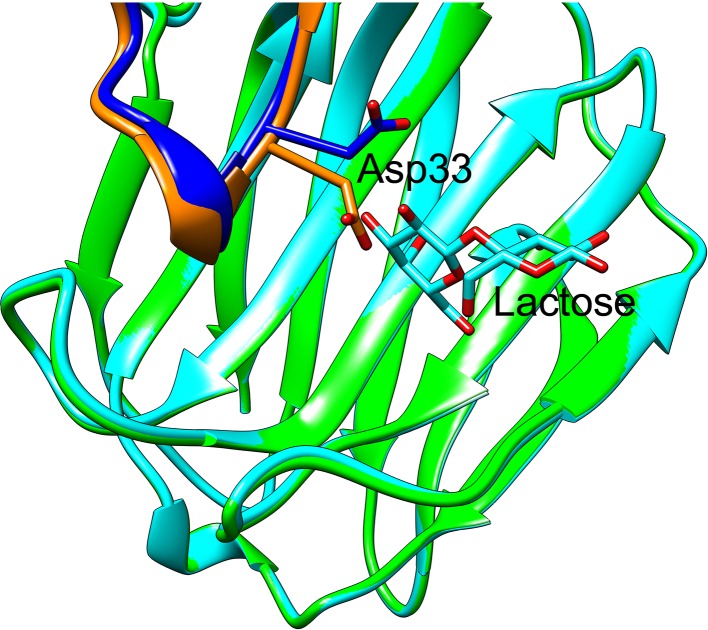
The overlay of the ligand binding sites of wild-type Gal-13 (PDB: 5XG7) and R53HH57R In the ligand binding sites of wild-type Gal-13, Asp33 (labeled by gold) from another asymmetric unit directly occupies a large area of the ligand binding site. However, lactose binding to R53HH57R could repel Asp33 (labeled by blue) and make this residue in awkward conformation.

All three other variants (R53HH57R, R53HR55NH57R, and R53HR55NR57RD33G) co-crystallized with lactose, but not Tris, even when the crystallization conditions contained 100 mM Tris. This indicates that these three variants prefer to binding lactose. These variants also contain the same two mutations (i.e. R53 to H and H57 to R), suggesting that arginine at position 57 is critical for Gal-13 binding lactose. Without this residue, Gal-13 cannot bind lactose. We merged the structures of the three variants with Gal-3 and Gal-8N. Lactose in the ligand binding sites of the three variants has similar conformations as lactose in Gal-3 and Gal-8N (Figure 3A). However, the pyran surface of the galactose residue in different structures could not be totally merged with each other, and glucose residues showed variable conformations (Figure 3B). The conserved tryptophan and histidine residues in the three variants showed the same conformation and position as in Gal-3 and Gal-8N. However, two other residues showed relatively variable conformations (Figure 3B). Arg57 in R53HH57R and R53HH57RD33G showed two different conformations. Arg57 in one conformation could indirectly stabilize the pyran ring of galactose through a water molecule (W) (Figure 4A). As in the ligand binding site of Gal-8N, Arg69 could directly make a cation–π interaction with the pyran ring (Figure 4B) [33]. The position of the water molecule (W) could perfectly overlay with a nitrogen atom of the side chain guanidinium group. But the distance between the water molecule and the pyran ring is longer than the distance between guanidinium group of Gal-8 Arg69 and the pyran ring. This implies the ligand binding in these variants binds lactose more weakly than it does in Gal-8N. In the crystal of wild-type Gal-13, Asp33 from one monomer occupies a large part of the ligand binding site of another. However, in the R53HH57R variant, lactose repealed the side chain of Asp33, making Asp33 adopt an awkward conformation (Figure 5, Supplementary Figure S2).

### Sugar binding assays of Gal-13 variants

**Figure 6:**
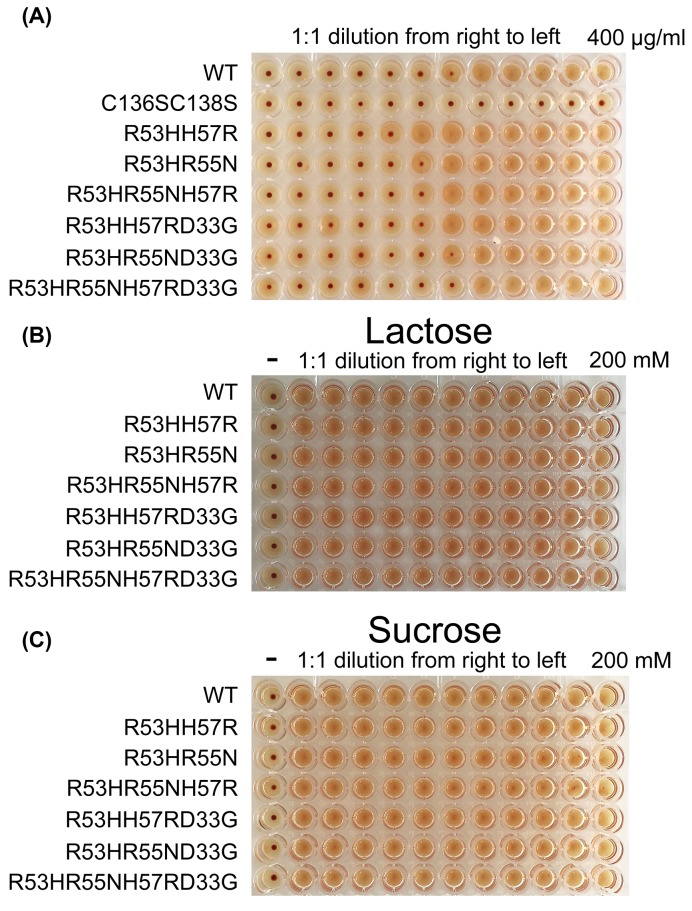
The hemagglutination assay (**A**) Except C136SC138S, the bio-activities of all variants are similar to wild-type Gal-13. C136SC138S, which exists as monomer, could not induce chicken erythrocyte agglutination. This indicates that the homodimerization of Gal-13 is critical for Gal-13 inducing hemagglutination. (**B, C**) 200 mM lactose and sucrose could not inhibit the hemagglutination reaction induced by Gal-13 variants.

Bio-activities of Gal-13 variants were functionally assessed using the hemagglutination assay. Wild-type Gal-13 induced hemagglutination at a minimum agglutination concentration (MAC) of 25 µg/ml (Figure 6A), consistent with previous reports [9]. With the exception of double mutant C136SC138S, we found that all other variants also promoted chicken erythrocyte agglutination. In the structure of wild-type Gal-13, Cys136, and Cys138 form two disulfide bonds between monomers in the dimer. Gel filtration showed that C136SC138S exists as a monomer, and thus probably could not induce hemagglutination. In our previous report [9], we tried to use DTT to reduce the disulfide bonds and inhibit hemagglutination. However, DTT (even at 10 mM) could not totally inhibit agglutination. Gel filtration showed that DTT did not completely reduce the disulfide bonds. Therefore, the remaining amount of dimeric Gal-13 still could promoted agglutination. Variant R53HH57R demonstrated greater bio-activity (MAC: 6.25 µg/ml) than wild-type Gal-13, and all other variants had similar activities (25 or 12.5 µg/ml) as wild-type protein. Overall, the mutations within the ligand binding site of Gal-13 did not profoundly change the activities of the variants in terms of promoting hemagglutination.

**Figure 7:**
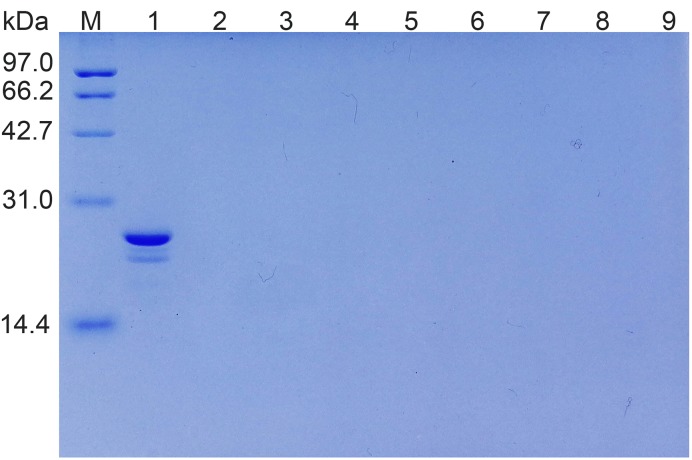
Solid phase assay. As a positive control, Gal-8_1-186 [33] could bind to lactose modified agarose beads (Lane 1) Lanes 2–9 indicate WT, C136SC138S, R53HR55N, R53HH57R, R53HR55NH57R, R53HH57RD33G, and R53HR55NH57RD33G could not bind to the beads.

We also tried to use lactose as an inhibitor of Gal-13-induced hemagglutination. However, even 200 mM lactose could not inhibit the effect (Figure 6B), consistent with our previous report [9]. As a negative control, sucrose also could not inhibit the reaction (Figure 6C). To determine whether Gal-13 variants could directly bind lactose, we performed a solid phase assay with lactose–Sepharose 6b (Figure 7). Whereas a significant amount of Gal-8_1-186 (21 kDa) (control) could bind to lactose-modified Sepharose 6b, SDS-PAGE showed that neither wild-type Gal-13 nor its variants were recovered from the lactose-modified column.

### Distribution of EGFP-tagged Gal-13 and the variants in Hela cells

**Figure 8:**
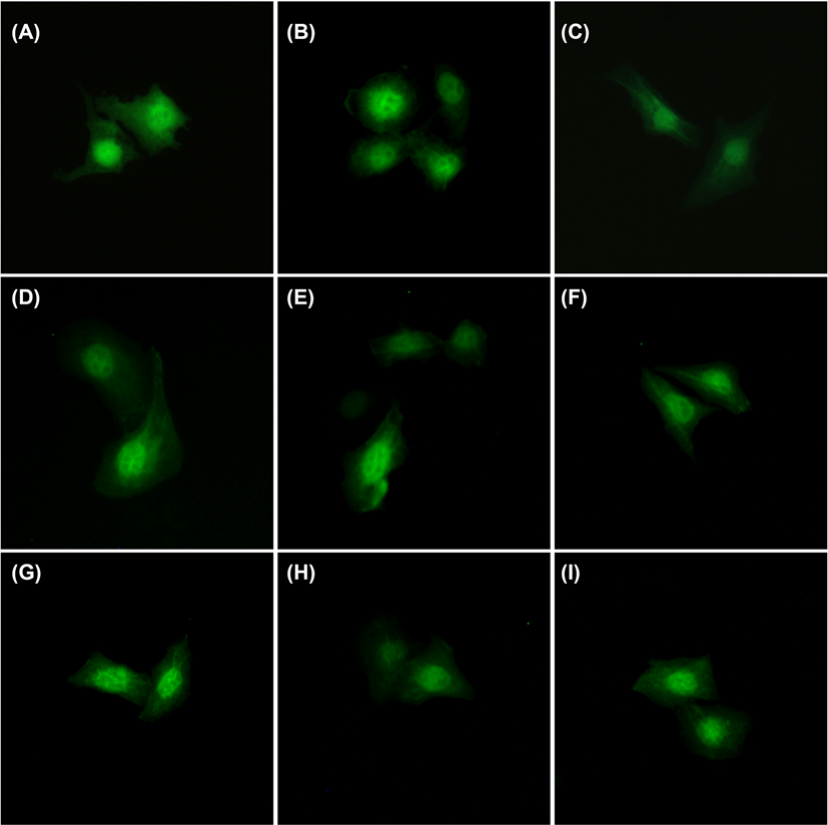
The distributions of EGFP-tagged Gal-13 variants in HeLa cell (**A**) WT. (**B**) C136SC138S. (**C**) R53H. (**D**) R53HR55N. (**E**) R53HR55ND33G. (**F**) R53HH57R. (**G**) R53HH57RD33G. (**H**) R53HH57RR55N. (**I**) R53HR55NH57RD33G. All Gal-13 variants are extensively distributed in nucleus which is similar as our previous report [9]. All Gal-13 variants are co-localized within filamentary materials, possibly actin.

To study the distribution of Gal-13 within HeLa cells, we produced EGFP N-terminal tagged Gal-13. Based on our crystallographic results, placing the tag at the N-terminus should not perturb Gal-13 dimer structure. Following 16 h transfection, we found EGFP-tagged wild-type Gal-13 was distributed in the nucleus and cytoplasm (Figure 8, Supplementary Figure S2), as previously reported [9]. In the nucleus, EGFP-tagged Gal-13 was not evenly distributed. Several areas showed high green fluorescence, whereas several others showed low green fluorescence. All variants showed similar distributions as wild-type protein in the cells. Several EGFP-tagged variants, for example R53H, clearly co-localized with filamentary material, possibly actin in the cells (Figure 8C). Because Than et al. and Balogh et al. used immunological methods to identify Gal-13, the lectin could be co-localized with actin [17,18]. If this were the case, then our results are consistent with this.

## Discussion

In our previous study on Gal-13 [9], we tried to co-crystallize the protein under conditions containing 50 mM lactose. However, we observed no electron density from the disaccharide within the ligand binding site. We also employed the hemagglutination assay, solid phase assay and biolayer interferometry to determine whether Gal-13 could even interact with lactose. All of these results were negative. The side chain of Arg53 in wild-type Gal-13, which protrudes from the bottom of the ligand binding site, did have a different conformation when compared with the conserved histidine in other galectins. Because we hypothesized that mutation of Arg53 to histidine would recover Gal-13’s ability to bind lactose, we generated a R53H variant and crystallized it under the same crystallization conditions as used for wild-type Gal-13. Once again, however, lactose electron density was absent. This indicates that further mutations are required to recover lactose binding. Moreover, this means that Gal-13 is missing several key residues necessary for lactose binding.

Upon aligning primary structures from all galectins [9], we found three residues (i.e. Arg53, Arg55, and His57) within the ligand binding site of Gal-13 that are different from other galectin–lactose binding sites. In the Gal-3 and Gal-8N CRDs, histidine, asparagine, and arginine occupy positions 53, 55, and 57 in Gal-13, respectively. The histidine residue (Gal-3 His 158 and Gal-8 His 65) at the corresponding position in Gal-3 and Gal-8N plays a key role in stabilizing lactose binding by establishing hydrogen bonds with O4 of lactose [34,35]. The conserved arginine residue (Gal-3 Arg162 and Gal-8 Arg69) is assumed to make a cation–π interaction with the pyran ring of the galactose residue [32,33]. Mutation of this arginine would greatly reduce the biological activity of Gal-8 [36]. Overall, in order to restore the ability of lactose to bind to Gal-13, we systematically modified the ligand binding site of Gal-13. The present results showed that double mutation of R53 to H and H57 to R was sufficient to recover the ability of Gal-13 to bind lactose. Crystal contacts indicate that Asp33 from an opposing monomer subunit occupies a relatively large area within the ligand binding site. However, Asp33 could not inhibit lactose binding to Gal-13. In fact, lactose binding to Gal-13 modifies the conformation of Asp33.

Based on the crystallographic studies of human galectins [8,9,32,33,37], we found, not surprisingly, that the ligand binding sites of galectins prefer to bind compounds containing hydroxyl groups. The carboxyl group of aspartate and glutamate actually constitute two partial hydroxyl groups. In the crystal structure of wild-type Gal-13, Asp33 from an opposing crystal contact monomer could take the place of the ligand binding site of the monomer [9]. In Gal-10, Glu33 from an opposing monomer also could occupy part of the ligand binding site [8]. Asp33 in Gal-13 and Glu33 in Gal-10 show quite similar conformations in their respective crystals. In Gal-2, Asp29 is directly inserted into the ligand binding site. Two hydroxyl groups from Asp29 occupy almost the same positions as O3 and O4 of lactose [37]. Note also that the CRDs of Gal-3, Gal-8N, Gal-10, and Gal-13 can bind glycerol. Glycerol is a flexible molecule that contains three hydroxyl groups. In Gal-3, Gal-8N, and Gal-10, the positions of the three hydroxyl groups from glycerol merge with O4, O5, and O6 of lactose [8,31,32]. We also mutated conserved residues in the Gal-10 ligand binding site; nevertheless, they could still bind glycerol [8]. Here, we found two Gal-13 variants (R53H and R53HR55N) that could co-crystallize with Tris, that also has three hydroxyl groups and shows a triangular shape when bound in the ligand binding site. However, the hydroxyl groups from Tris could not be merged with the oxygen atoms of lactose. Overall, it seems that the ligand binding sites of individual galectins have evolved as a trap for small hydroxylated compounds. This implies that the ligand binding site has the potential to bind other biomacromolecules.

In the hemagglutination assay, all ligand binding site variants could induce erythrocyte agglutination like wild-type Gal-13; however, lactose could not inhibit their bio-activities. Two reasons could explain this phenomenon. The first one is that Gal-13 induces erythrocyte agglutination not via the canonical ligand binding site. The second one is that Gal-13 could strongly bind unknown ligands on the erythrocyte cell membrane that lactose cannot effectively inhibit. In our previous hemagglutination assay with Gal-2 and Gal-10, we also observed this effect [8,37,38]. Both of them could induce chicken erythrocyte agglutination, but were not sensitive to lactose inhibition. The actual reason for this requires further study. In addition, all of our Gal-13 variants could also not bind lactose-modified Sepharose 6b, which seems contrary to the crystallographic studies where several variants could be co-crystallized with lactose. Nevertheless, the co-crystallization time (∼7–14 days) is much longer than the time required (∼2 h) for binding using the solid phase assay. This in turn implies that lactose binds to the variants in the crystals very slowly.

Galectin-13 was first isolated from human placenta which only contains ∼0.6% carbohydrate [10]. During pregnancy, Gal-13 is highly expressed in the placenta (specifically in the syncytiotrophoblast). By using pull-down and mass spectroscopy, Than et al. identified that Gal-13 could specifically bind to annexin II and actin [17]. Moreover, immunofluorescence also showed that Gal-13 could be co-locallized with actin in the syncytiotrophoblast and bewo cells [18]. This implies that Gal-13 secretion or transport may occur via these cytoskeleton proteins. Here, we found that EGFP-tagged Gal-13 and its variants also co-localize with several filamentary entities (Figure 8). If one of these were actin, then our results are consistent with previously published results. This also indicates that EGFP does not influence the distribution of Gal-13 and its variants. The C136SC138S mutant is a monomeric protein, but the EGFP-tagged protein still could be co-localized with a linear structure. This means that co-localization is independent of the global structure of Gal-13. With the exception of C136SC138S, all other variants have mutations within the ligand binding site. Nevertheless, all of them could also be co-localized with the filamentary molecules, indicating that binding is also independent of the ligand binding site. It would be interesting to investigate how Gal-13 interacts with these filamentary molecules. In addition, we also found that our variants are mainly concentrated within the nucleus. However, this distribution seems to be in dynamic flux. In some Hela cells, the green fluorescence was seen to be equally distributed, but in other cells, they were not. In the nucleus, Gal-10 is only distributed in euchromatin, but not in heterochromatin [38,39]. Gal-13 may also have the same behavior as with Gal-10. Previous immunohistochemistry data also showed that Gal-13 is highly concentrated in the nucleus. Overall, the distribution of EGFP-tagged Gal-13 variants is similar to previously published results [9].

In conclusion, we demonstrated here that Gal-13 cannot naturally bind lactose. Although the primary and tertiary/quarternary structures of Gal-13 is similar to all other galectins, Gal-13 appears to be a different galectin. Up to now, the molecular function of Gal-13 remains unknown, aside from it being related to pre-eclampsia. Future work should focus on identifying more physiological ligands of Gal-13 and to determine whether Gal-13 is a signaling transduction protein involved in regulating gene replication and/or transcription. Answers to these questions might help treat pre-eclampsia.

## Supporting information

**supplementary Figure:** 

**supplementary Figure:** 

**Supplemental Table:** 

## Abbreviations

CRD: carbohydrate-binding domain
EGFP: enhanced green fluorescent protein
Gal: galectin
DMEM: Dulbecco’s modified Eagle’s medium
MAC: minimum agglutination concentration
PBS: phosphate-buffered saline
SDS-PAGE: sulfate-polyacrylamide gel electrophoresis
