# Supplementary material for: Resetting the ligand binding site of placental protein 13/galectin-13 recovers its ability to bind lactose
Source: Biosci Rep. 2018 Dec 14;38(6):BSR20181787. doi: 10.1042/BSR20181787 (PMC6294630; doi:10.1042/BSR20181787)
Supplement: Supplementary file 1 [file bsr20181787_Supp1.pdf]

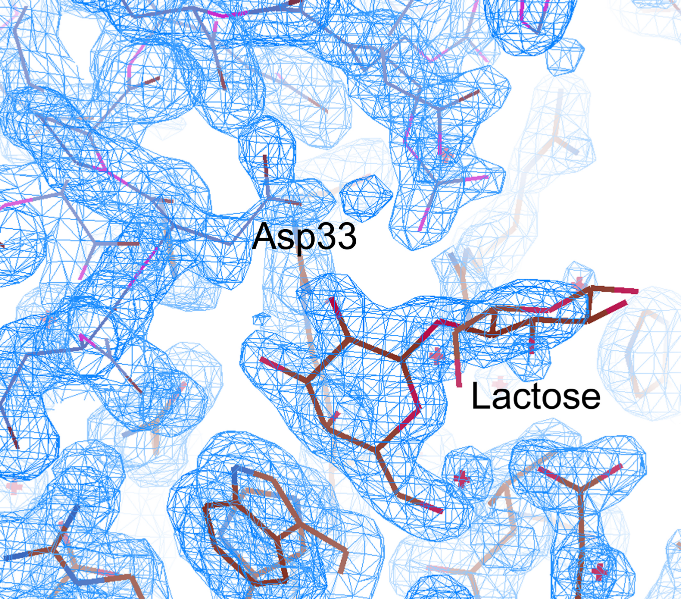
Asp33
Lactose

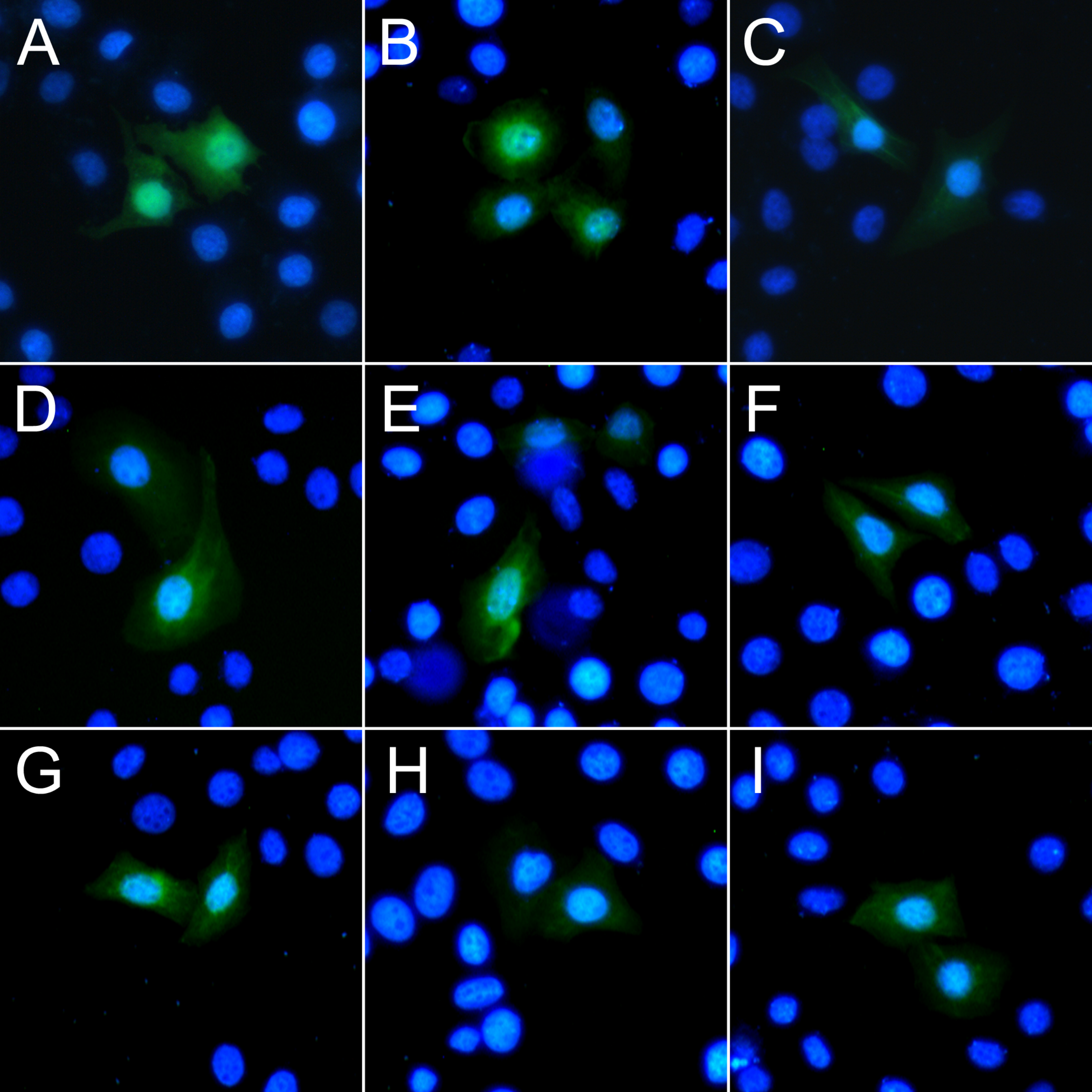
A
B
C
D
E
F
G
H
I
